# Agreement between planned, robot-validated, and postoperative radiographic posterior tibial slope in ROSA-assisted total knee arthroplasty

**DOI:** 10.1097/MD.0000000000049767

**Published:** 2026-07-17

**Authors:** Ramadan Ozmanevra, Baris Polat, Ozenc Altinoz, Nihat Demirhan Demirkiran, Tolgay Karanfiller

**Affiliations:** a Faculty of Medicine, Department of Orthopedics and Traumatology, Near East University, Nicosia, Cyprus; b Faculty of Medicine, Department of Orthopedics and Traumatology, Primemed Clinic, Kyrenia, Cyprus; c Faculty of Medicine, Department of Orthopedics and Traumatology, Kütahya Health Sciences University, Kütahya, Turkey; d Faculty of Education, Cyprus International University, Nicosia, Cyprus.

**Keywords:** posterior tibial slope, radiographic agreement, robotic-assisted total knee arthroplasty, ROSA, sagittal alignment

## Abstract

Posterior tibial slope (PTS) is a key determinant of sagittal knee mechanics, cruciate ligament loading, and flexion-extension behavior after total knee arthroplasty (TKA). Accurate reproduction of the intended PTS is therefore an important objective in contemporary TKA, particularly when patient-specific alignment targets are used. The aim of this study was to evaluate the radiographic agreement among planned, robot-validated, and postoperative PTS values in ROSA-assisted TKA using standardized true lateral radiographs and blinded repeated measurements, and to determine the reliability of postoperative radiographic measurements. This retrospective observational radiological study, reported in accordance with the STrengthening the Reporting of OBservational studies in Epidemiology statement, included knees that underwent robotic-assisted primary TKA using the ROSA system and had complete intraoperative robotic records and adequate postoperative true lateral radiographs. Postoperative true lateral radiographs were obtained at 6-week follow-up. Planned PTS values were obtained from the robotic planning interface, and final robot-validated PTS values were recorded intraoperatively after tibial preparation. Postoperative PTS was measured on standardized true lateral radiographs by 2 independent blinded orthopedic surgeons, each performing repeated measurements at a 2-week interval. Agreement was assessed using paired comparisons, mean absolute differences, outlier analysis, and Bland–Altman evaluation. A total of 73 knees from 64 patients were initially reviewed, and 72 knees with complete paired measurements were included in the final analysis. There was no statistically significant difference between planned and robot-validated PTS values. The mean absolute difference between planned and robot-validated PTS was 0.57°, and 80.6% of knees were within ±1° of the planned target, while all knees were within ±2° and ±3°. Similarly, no statistically significant difference was observed between robot-validated and postoperative mean radiographic PTS values. Radiographic measurement reliability was excellent, with ICC(3,1) = 0.983 (95% confidence interval = 0.976–0.989) and ICC(3,*k*) = 0.996 (95% confidence interval = 0.994–0.997). ROSA-assisted TKA demonstrated close radiographic agreement among planned, robot-validated, and postoperative PTS values, with low absolute deviations and excellent measurement reliability. Because this study lacked a comparator group and did not assess clinical outcomes, these findings should be interpreted as evidence of radiographic reproducibility rather than evidence of superiority over conventional instrumentation or proof of clinical benefit.

## 1. Introduction

Posterior tibial slope (PTS) is a critical determinant of knee kinematics, sagittal balance, and cruciate ligament loading.^[1–7]^ Variations in PTS influence tibial translation, flexion-extension mechanics, and ligament tension, thereby affecting both functional behavior and implant biomechanics after total knee arthroplasty (TKA).^[5–10]^ Accurate reproduction of the intended PTS has therefore become an important goal in contemporary knee arthroplasty practice.^[1–4,11]^

Native PTS demonstrates substantial interindividual variability and is influenced by compartmental anatomy, ethnicity, imaging modality, and measurement technique.^[1–4]^ Several morphologic studies have shown that medial and lateral tibial slopes may differ within the same knee and that reported values vary according to the selected reference axis and imaging method.^[1–4]^ These observations support the concept that slope targets should be individualized rather than universally fixed.^[1–4]^ Population-specific studies from Turkish cohorts further indicate that tibial morphology and sagittal alignment parameters may differ across populations, emphasizing the importance of patient- and population-adapted planning.^[12–16]^

Conventional instrumentation in TKA has inherent limitations in controlling sagittal alignment, particularly in PTS.^[11]^ Even small deviations during tibial resection may lead to clinically meaningful changes in knee mechanics, especially in cruciate-retaining or cruciate-substituting constructs.^[5–7]^ Robotic-assisted TKA systems have therefore been developed to improve the precision and reproducibility of bone preparation by integrating digital planning, intraoperative feedback, and real-time validation.^[11]^

Robotic platforms allow surgeons to define patient-specific alignment targets and quantitatively verify achieved component positioning during surgery.^[11]^ Previous studies have reported high accuracy of robotic systems in both coronal and sagittal alignment, although postoperative radiographic measurements remain susceptible to variability related to imaging quality, axis selection, rotational malposition, and observer interpretation.^[11,17–20]^ For that reason, assessment of robotic accuracy should not rely solely on postoperative imaging, but should also examine the relationship between planned targets, intraoperative robotic execution, and postoperative radiographic verification.^[11,17–19]^

While previous ROSA (Zimmer Biomet, Warsaw)-assisted TKA studies have evaluated general alignment accuracy, less attention has been directed toward agreement among planned, intraoperatively robot-validated, and postoperative radiographic PTS values using standardized true lateral radiographs and blinded repeated measurements. The specific contribution of this study is its focused assessment of PTS reproducibility across 3 stages of the ROSA workflow: preoperative/intraoperative planning, intraoperative robot validation, and postoperative radiographic measurement. The aim of this study was to evaluate the radiographic agreement among planned, robot-validated, and postoperative PTS values in ROSA-assisted TKA using standardized true lateral radiographs and blinded repeated measurements. We hypothesized that robotic-assisted TKA would demonstrate close radiographic agreement between planned, intraoperative, and postoperative slope values, with low absolute deviation, low radiographic outlier rates, and excellent reliability of postoperative radiographic measurements.^[11,17–19]^

## 2. Materials and methods

### 2.1. Study design and patient population

This retrospective observational radiological study was reported in accordance with the STrengthening the Reporting of OBservational studies in Epidemiology statement and included patients who underwent robotic-assisted primary TKA using the ROSA robotic system (Zimmer Biomet). A total of 73 knees from 64 patients were initially reviewed. Knees were eligible if complete robotic planning and intraoperative validation data were available and if adequate postoperative true lateral radiographs had been obtained. Knees with incomplete robotic records, missing paired slope data, or postoperative radiographs not meeting predefined quality criteria were excluded. One knee was excluded because complete paired slope data were unavailable. After exclusion, 72 knees with complete paired measurements were included in the final analysis.

The study cohort consisted of 27 male and 37 female patients with a mean age of 68.5 ± 8.2 years (range, 46–86 years) and a mean body mass index of 29.3 ± 5.2 kg/m^2^ (range, 20.5–48.1). Fifty-five patients underwent unilateral TKA, and 9 patients underwent bilateral procedures. Ethical approval was obtained from the Cyprus International University Ethics Committee, Cyprus International University, Nicosia, Cyprus (approval number: EKK24-25/07/07; approval date: April 4, 2025). All procedures were conducted in accordance with the Declaration of Helsinki.

### 2.2. Surgical technique and robotic planning

All procedures were performed by a single arthroplasty surgeon who had completed formal ROSA Knee System training before the study period, using a standardized medial parapatellar approach and the Zimmer Biomet Vanguard Knee System with a posterior-stabilized design. Before planning this study, the surgeon had performed 50 ROSA-assisted TKA procedures. No case required conversion to conventional instrumentation. Pre-resection planning was performed using the robotic platform to define patient-specific alignment targets, including PTS.^[11]^ The planned PTS was determined using the ROSA Knee System planning workflow and adjusted according to implant design, ligament status, and intraoperative gap assessment. For the purposes of analysis, the planned PTS was defined as the slope value recorded during robotic planning.

During surgery, tibial bone preparation was performed under robotic guidance. After tibial resection, the robot-validated PTS was defined as the final accepted intraoperative slope value recorded by the robotic system after completion of tibial preparation and robotic validation.^[11]^ This value was used as the intraoperative reference for robotic execution accuracy.

### 2.3. Radiographic evaluation

Postoperative PTS was assessed on standardized true lateral knee radiographs obtained at 6-week follow-up using a standardized radiographic protocol. Radiographs were included only if posterior femoral condylar overlap was ≤6 mm in order to minimize rotational measurement error, as previously recommended.^[17]^ Two independent orthopedic surgeons, blinded to all robotic data, evaluated all radiographs. Each observer performed measurements twice at a 2-week interval.

PTS was defined as the angle between the tangent to the tibial component surface and a line drawn perpendicular to the longitudinal tibial shaft axis on the true lateral radiograph.^[18–20]^ The mean value of the repeated blinded postoperative measurements was used as the principal postoperative radiographic PTS value for statistical comparison with robotic data.

### 2.4. Outcomes and statistical analysis

The primary outcome was the agreement between planned PTS and robot-validated PTS. The secondary outcome was the agreement between robot-validated PTS and postoperative mean radiographic PTS. Additional descriptive measures included mean absolute deviation, median absolute deviation, and the proportion of knees falling within predefined outlier thresholds of ±1°, ±2°, and ±3°.

Continuous variables were expressed as mean ± standard deviation (SD) with corresponding 95% confidence intervals (CIs). Paired-samples *t* tests were used to compare planned PTS versus robot-validated PTS and robot-validated PTS versus postoperative mean PTS. Because statistical nonsignificance alone does not fully characterize surgical agreement, mean absolute difference, median absolute difference, outlier analysis, and Bland–Altman evaluation were also performed. Measurement reliability of postoperative radiographic PTS assessment was evaluated using intraclass correlation coefficients (ICCs) based on a 2-way mixed-effects model with absolute agreement. Single-measure reliability was reported as ICC(3,1), and average-measure reliability across repeated observer measurements was reported as ICC(3,*k*), with corresponding 95% CIs. Statistical significance was set at *P* < .05.

## 3. Results

### 3.1. Patient characteristics

A total of 73 knees from 64 patients were initially reviewed. One knee was excluded because complete paired slope data were unavailable. Complete paired measurements were available for 72 knees, which constituted the final analytic cohort. Patient demographics and perioperative characteristics are summarized in Table 1.

**Table 1:** Patient demographics and perioperative characteristics.

Variable	Value
Number of patients	64
Number of knees	73
Knees included in paired analysis	72
Sex (male/female)	27/37
Age (yr), mean ± SD (range)	68.5 ± 8.2 (46–86)
Body mass index (kg/m^2^), mean ± SD (range)	29.3 ± 5.2 (20.5–48.1)
Unilateral TKA, n (%)	55 (85.9%)
Bilateral TKA, n (%)	9 (14.1%)
Robotic system	ROSA (Zimmer Biomet)

SD = standard deviation, TKA = total knee arthroplasty.

### 3.2. Planned versus robot-validated PTS

**Figure 1.**
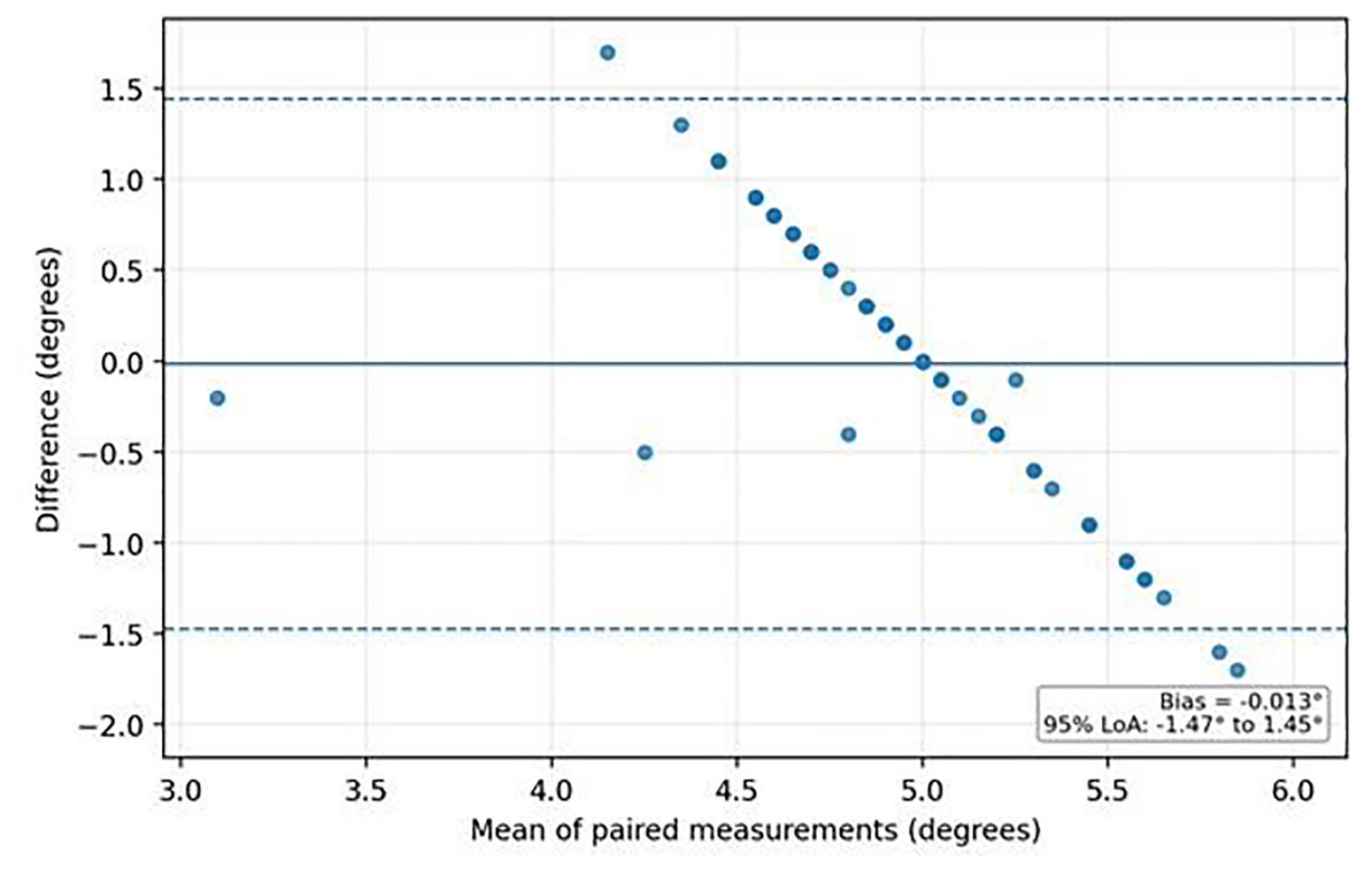
Bland–Altman plot: planned versus robot-validated PTS. PTS = posterior tibial slope.

Comparison between planned PTS and robot-validated PTS demonstrated no statistically significant difference. The mean paired difference was −0.0125° (SD = 0.7447), with a 95% CI ranging from −0.1875° to 0.1625°, and this difference was not statistically significant (*t*(71) = −0.142, *P* = .887). The mean absolute difference for this comparison was 0.57°, and the median absolute difference was 0.45°. A total of 58 of 72 knees (80.6%) were within ±1° of the planned target, while all 72 knees (100%) were within both ±2° and ±3° (Table 2). Bland–Altman analysis showed a mean bias of −0.0125°, with 95% limits of agreement from −1.47° to 1.45°. These findings indicate minimal directional bias and a narrow distribution of error around the planned target (Fig. 1).

**Table 2:** Agreement between planned and robot-validated PTS.

Comparison	Mean diff (°)	SD	95% CI	*t*	df	*P*	Mean abs diff (°)	Within ±1°	Within ±2°/±3°
Planned PTS – robot-validated PTS	−0.0125	0.7447	−0.1875 to 0.1625	−0.142	71	.887	0.57	58/72 (80.6%)	72/72 (100%)/72/72 (100%)

CI = confidence interval, PTS = posterior tibial slope, SD = standard deviation.

### 3.3. Robot-validated versus postoperative mean PTS

**Figure 2.**
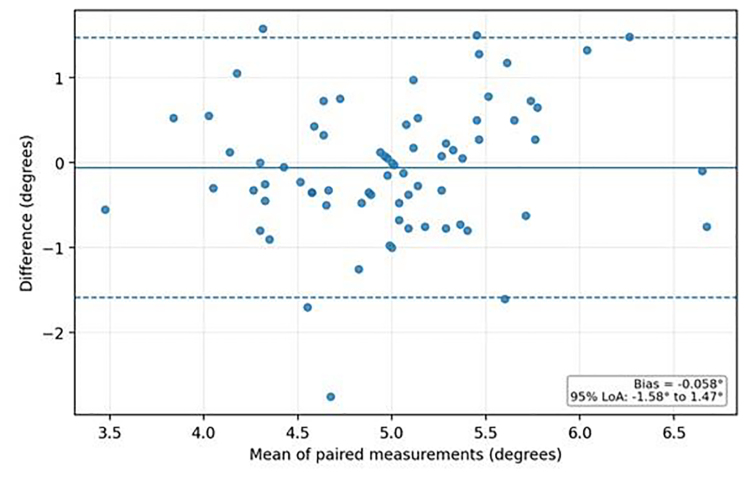
Bland–Altman plot: robot-validated versus postoperative mean PTS. PTS = posterior tibial slope.

Comparison between robot-validated PTS values and postoperative mean radiographic PTS values also demonstrated no statistically significant difference. The mean paired difference was −0.0583° (SD = 0.7789), with a 95% CI of −0.2414° to 0.1247°, and this difference was not statistically significant (*t*(71) = −0.635, *P* = .527). The mean absolute difference for this comparison was 0.60°, and the median absolute difference was 0.49°. A total of 61 of 72 knees (84.7%) were within ±1° of the robot-validated value, 71 of 72 knees (98.6%) were within ±2°, and all 72 knees (100%) were within ±3° (Table 3). Bland–Altman analysis demonstrated a mean bias of −0.0583°, with 95% limits of agreement from −1.58° to 1.47° (Fig. 2). This again indicated low systematic bias and acceptable dispersion between intraoperative robotic and postoperative radiographic measurements.

**Table 3:** Agreement between robot-validated and postoperative mean radiographic PTS.

Comparison	Mean diff (°)	SD	95% CI	*t*	df	*P*	Mean abs diff (°)	Within ±1°	Within ±2°/±3°
Robot-validated PTS – postoperative mean PTS	−0.0583	0.7789	−0.2414 to 0.1247	−0.635	71	0.527	0.60	61/72 (84.7%)	71/72 (98.6%)/72/72 (100%)

CI = confidence interval, PTS = posterior tibial slope, SD = standard deviation.

### 3.4. Reliability of postoperative radiographic measurements

Reliability analysis of postoperative radiographic PTS measurements demonstrated excellent agreement. The ICC for single measurements was ICC(3,1) = 0.983 (95% CI = 0.976–0.989), while the average-measure reliability was ICC(3,*k*) = 0.996 (95% CI = 0.994–0.997), indicating excellent repeatability and reproducibility of the radiographic measurement technique (Table 4).

**Table 4:** Reliability of postoperative PTS measurements.

ICC model	ICC value	95% CI
ICC(3,1) – single measure	0.983	0.976–0.989
ICC(3,*k*) – average measure	0.996	0.994–0.997

ICCs were calculated using a 2-way mixed-effects absolute-agreement model. ICC(3,1) denotes single-measure reliability, and ICC(3,*k*) denotes average-measure reliability across repeated observer measurements.

CI = confidence interval, ICC = intraclass correlation coefficient, PTS = posterior tibial slope.

## 4. Discussion

The principal finding of this study is that ROSA-assisted TKA demonstrated close radiographic agreement among planned, intraoperatively validated, and postoperative radiographic PTS values.^[11]^ Planned and robot-validated slopes showed minimal deviation, and postoperative radiographic measurements remained highly consistent with intraoperative robotic values. Importantly, the present analysis extends beyond simple significance testing by demonstrating low mean absolute deviation, a low burden of radiographic outliers, and minimal Bland–Altman bias. Taken together, these findings support the ability of robotic-assisted TKA to reproducibly execute surgeon-defined sagittal alignment targets.^[11]^

The absence of a statistically significant difference between planned and robot-validated PTS values indicates that the robotic platform was able to translate surgical intent into intraoperative execution with a high degree of fidelity.^[11]^ However, the present findings are better interpreted in terms of agreement rather than mere absence of statistical difference. The mean absolute error of 0.57° and the finding that 80.6% of knees were within ±1° of the target, with all knees within ±2°, indicate that deviation from the planned slope was not only statistically negligible but also small in practical terms. This pattern is consistent with previous reports showing high precision of robotic systems in alignment control and bone preparation during TKA.^[11]^

Similarly, postoperative mean radiographic PTS showed close agreement with the robot-validated intraoperative value. The mean absolute difference of 0.60°, the 84.7% rate within ±1°, and the 98.6% rate within ±2° suggest that standardized radiographic assessment can provide reproducible postoperative verification of intraoperative robotic execution.^[17–19]^ This point is important because postoperative measurement of PTS is known to be influenced by lateral radiograph quality, tibial axis definition, and observer-dependent interpretation.^[17–20]^ The low bias observed on Bland–Altman analysis supports the conclusion that, under standardized imaging conditions, postoperative radiographs did not systematically overestimate or underestimate achieved slope in this cohort.^[17–19]^ These observations are directionally consistent with a recent study by Ji et al, who reported that computer-assisted navigation achieved smaller tibial sagittal (slope) deviation than conventional TKA (1.7° ± 1.1° vs 2.3° ± 1.4°), together with fewer mechanical axis outliers and modestly better early postoperative recovery, supporting the broader premise that technology-assisted TKA can improve the reproducibility of alignment execution even when the study design differs from the present plan-versus-execution analysis within a robotic workflow.^[12]^

PTS plays a crucial role in knee biomechanics, sagittal balance, and cruciate ligament loading.^[5–10]^ Excessive posterior slope may increase anterior tibial translation and alter ligament tension, whereas insufficient slope may adversely affect flexion mechanics and posterior stability depending on implant design and ligament status.^[5–7]^ For that reason, accurate and reproducible execution of the intended slope remains relevant even when the precise optimal target varies according to patient anatomy, implant philosophy, and surgeon preference.^[1–7]^ The present data indicate that robotic assistance can help maintain this intended sagittal target with a low error burden.^[11]^

Population-specific morphology provides additional context for these findings. Several studies have demonstrated that tibial slope varies according to ethnicity, sex, compartment, and measurement technique.^[1–4,13–15]^ Turkish cohorts have shown distinctive proximal tibial morphologic characteristics, and implant sizing differences have also been reported in Turkish TKA populations.^[13–16]^ In addition, metaphysio-diaphyseal relationships may influence sagittal alignment interpretation, particularly in osteoarthritic knees with atypical proximal tibial geometry.^[20,21]^ In that setting, the ability of a robotic platform to reproduce a deliberately chosen, patient-specific slope may be particularly valuable.^[11,13–16,20,21]^

From a methodological standpoint, the study has several strengths. First, it examined the full chain of slope execution, including planned target, intraoperative robotic validation, and postoperative radiographic verification.^[11]^ Second, postoperative imaging quality was standardized by restricting inclusion to true lateral radiographs with posterior femoral condylar overlap ≤6 mm, thereby reducing rotational measurement error.^[17]^ Third, radiographic measurements were performed by 2 blinded orthopedic surgeons with repeated assessments, and reliability analysis showed excellent measurement consistency. These methodological features strengthen confidence that the observed agreement was not merely an artifact of imprecise measurement.

This study has several limitations. First, it was retrospective and conducted at a single center using a single robotic platform. Second, there was no comparator group treated with conventional instrumentation or another robotic platform; therefore, comparative superiority cannot be inferred. Third, this study intentionally focused on PTS and did not evaluate coronal alignment, femoral or tibial rotation, joint-line restoration, or soft-tissue balancing parameters. Therefore, the findings should not be interpreted as a comprehensive assessment of overall component positioning accuracy in ROSA-assisted TKA. Fourth, postoperative slope assessment was based on 2-dimensional radiographs rather than 3-dimensional imaging, and radiographic measurements remain inherently sensitive to axis selection and projection quality despite strict standardization.^[17–20]^ Fifth, PROMs, range of motion, survivorship, gait analysis, and biomechanical outcomes were not evaluated; therefore, the clinical relevance of small radiographic deviations cannot be determined. Finally, all procedures were performed by a single surgeon, and although formal ROSA training had been completed before the study period, a potential learning-curve effect cannot be fully excluded.

## 5. Conclusion

ROSA-assisted TKA demonstrated close radiographic agreement among planned, robot-validated, and postoperative PTS values, with low absolute deviations and excellent measurement reliability. These findings support the radiographic reproducibility of PTS execution using the ROSA workflow and indicate that standardized true lateral radiographs can provide dependable postoperative confirmation of the achieved tibial slope. However, because this study lacked a comparator group and did not assess clinical outcomes, the findings should not be interpreted as evidence of superiority over conventional instrumentation or as proof of clinical benefit.

## Author contributions

**Conceptualization:** Ramadan Ozmanevra, Baris Polat, Ozenc Altinoz, Nihat Demirhan Demirkiran.

**Data curation:** Ramadan Ozmanevra, Baris Polat.

**Methodology:** Ramadan Ozmanevra, Baris Polat, Ozenc Altinoz.

**Supervision:** Ramadan Ozmanevra, Baris Polat, Ozenc Altinoz, Nihat Demirhan Demirkiran, Tolgay Karanfiller.

**Writing – original draft:** Ramadan Ozmanevra, Baris Polat, Ozenc Altinoz, Nihat Demirhan Demirkiran.

**Writing – review & editing:** Ramadan Ozmanevra, Baris Polat.

**Formal analysis:** Ozenc Altinoz, Nihat Demirhan Demirkiran, Tolgay Karanfiller.
